# Conformational characterization of a novel anti-HER2 candidate antibody

**DOI:** 10.1371/journal.pone.0215442

**Published:** 2019-05-09

**Authors:** Leina Moro Pérez, Azalia de la Caridad Rodríguez Taño, Lázaro Roberto Martín Márquez, Jose Alberto Gómez Pérez, Aisel Valle Garay, Rancés Blanco Santana

**Affiliations:** 1 Department of Process Development, Center of Molecular Immunology, Havana, Cuba; 2 Department of Quality Control, Center of Molecular Immunology, Havana, Cuba; 3 Faculty of Biology, Protein Study Center, Havana, Cuba; Consiglio Nazionale delle Ricerche, ITALY

## Abstract

Regulatory agencies establish that a broad physicochemical and biological characterization is necessary for the evaluation of comparability between a biosimilar candidate product and a reference commercial drug. Between them, conformational characterization of proteins is of vital importance to determine its folding and biological functions. In this work, the conformational features of a novel monoclonal antibody (called 5G4) were evaluated by means of circular dichroism spectroscopy and fluorescence. Secondary structure and thermal stability of mAbs were determined by circular dichroism in the far ultraviolet, while three-dimensional folding of proteins was analyzed by both circular dichroism in the near ultraviolet and intrinsic tryptophan fluorescence. In all experiments, Herceptin (Roche) was used as control. Both antibodies showed a composition of secondary structure predominantly of β-sheets (55–56%) and thermal stability of ~ 75°C, suggesting structural similarity. The three-dimensional folding of proteins was also similar due to the absorption spectra of the aromatic residues and the emission wavelength maxima by fluorescence were comparable. The values of the fluorescence attenuation constant (Stern-Volmer constant) for increasing concentrations of acrylamide were also similar, suggesting a degree of exposure of tryptophan residues similar, although it was slightly decreased for Herceptin. Our data permit to consider that 5G4 monoclonal antibody showed similar conformational characteristics when compared with Herceptin.

## Introduction

Despite significant advances in the diagnosis and treatment of cancer, this disease remains one of the leading causes of morbidity and mortality in the world [1]. Breast cancer is the most common cause of cancer-related deaths in women, comprising almost a third of all malignancies in females [2]. The heterogeneous nature of breast cancer has implications for both patients and medical research. For this reason, treatment strategies are directed towards molecular markers [3]. Currently, one of the major advances in this therapy has been the introduction of monoclonal antibodies (mAbs) targeting specific molecules overexpressed during tumorigenesis (e.g. growth factor receptors) [4].

Trastuzumab (Herceptin, Roche) is a humanized mAb directed toward the epidermal growth factor receptor type 2 (HER2 or HER-2/neu), which is overexpressed in 15 to 30% of invasive breast adenocarcinomas [5]. It was the first mAb approved in 1998 by the Food and Drug Administration of the United States of America (FDA) and also by the European Medicines Agency (EMA) in 2000 for the treatment of breast cancer patients with advanced or metastatic disease [6, 7]. Therapeutic schemes using Herceptin significantly provide clinical benefits in treated patients [6, 7].

Although effective, Herceptin based therapies are still expensive. In fact, the national health system of Cuba acquires this product at a high price in the international market (US $ 30,000 the treatment of one patient per year), which means that the number of patients who can obtain this benefit is limited [8]. The approval of biosimilar molecules has been recognized not only as an alternative, but also as a necessity to increase health coverage and improve the quality of life of cancer patients [9].

The assessment of comparability between a novel biological product and an innovator drug comprises both physicochemical and biological characterization; predicting if certain characteristics of products are similar [10–12]. The structural elements of a protein determine its function; therefore, it is important to assess the higher order structure of novel biological products. Specifically, the conformational characterization of proteins provide evidences regarding the state of their folding, their stability and participation in biological processes [13, 14].

Taking into account that Herceptin production patent expired, the Center of Molecular Immunology (Havana, Cuba) developed a novel mAb against the HER2 molecule (known as 5G4) as a potential tool for the treatment of tumors that overexpress this receptor. In the present work, it is shown a conformational characterization by circular dichroism and fluorescence spectroscopy of 5G4 mAb using Herceptin as reference product.

## Materials and methods

### Monoclonal antibodies

Herceptin (Roche, Switzerland) is composed of 1328 amino acids in its primary sequence with an average molecular mass of 148.3 KDa, due to the presence of N-oligosaccharides. Like other IgG1 molecules, it contains a biantennary oligosaccharide linked to nitrogen present in the asparagine residue conserved in position 300, enclosed between the CH2 domains [15]. Herceptin is produced by mean of the Chinese Hamster Ovary cells (CHO) expression system and marketed as a freeze-dried 150 mg of Trastuzumab. The lyophilized formulation was reconstituted with sterile water for injection to a concentration of 21 mg/mL of Trastuzumab and subsequently diluted with the formulation solution to a final concentration of 10 mg/mL.

**Fig 1:**
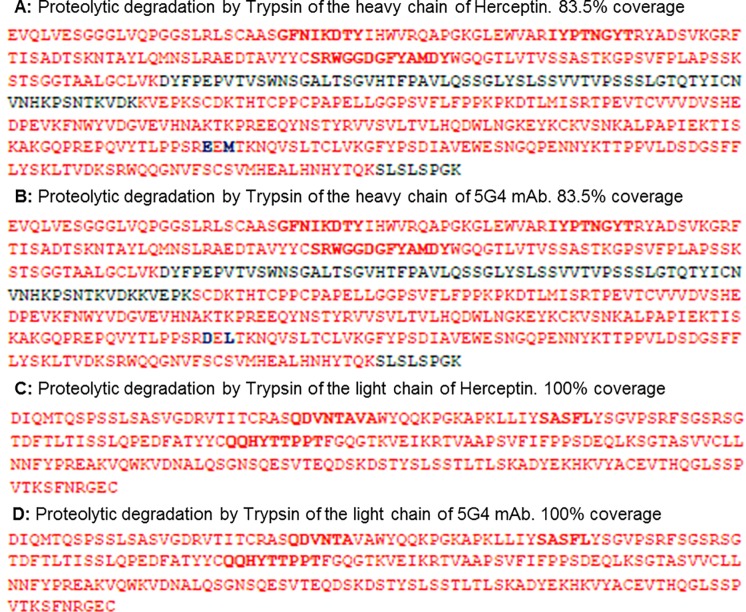
Sequence by MALDI and ESI of Herceptin and 5G4 mAb. The proteolytic degradation of the heavy chain and the light chain for both antibodies is observed. In red: mapped sequence, in black: sequence without mapping and highlighted in red: the complementarity determining regions. A and B: Proteolytic degradation by Trypsin of the heavy chain of Herceptin and 5G4 mAb, respectively. C and D: Proteolytic degradation by Trypsin of the light chain of Herceptin and 5G4 mAb, respectively.

The 5G4 mAb was generated based on the sequence of the original antibody (Herceptin) (Fig 1 and S1 Fig) and the non-secreting murine myeloma cells (NS0) expression system was used.

The formulation of 5G4 mAb (similar to Herceptin) was L-histidine (0.3 mg/mL), L-histidine-HCl (0.460 mg/mL), trehalose dihydrate (18.92 mg/mL) and polysorbate 20 (0.083 mg/mL) and the final concentration of this mAb was 10 mg/mL.

Three lots of final product of 5G4 mAb and two lots of the Herceptin were used.

### Molecular exclusion chromatography

The mAbs were desalted by a change of pH buffer with exclusion chromatography using a 5 mL HiTrap column equilibrated with 20 mmol/L PBS at pH 7.4. An ÄKTAprime plus (GE Healthcare, USA) type FPLC (Fast Performance Liquid Chromatography) chromatographic system following the manufacturer's recommendations. Then, 500 μL of each mAb formulation were injected at a flow rate of 3 mL/min and fractions of 500 μL were collected. The chromatographic process was monitored by absorbance readings at 280 nm, conductivity and pH values. The results were analyzed with the program Origin v8.0 (OriginLab Corporation, USA).

### Determination of protein concentration

Protein concentration in PBS was determined by reading the absorbance at 280 nm from the absorption spectrum in a range of 200 to 350 nm with dispersion correction at 350 nm. A double beam spectrophotometer model Jasco V-730 (Jasco Corporation, Japan) and quartz cuvettes of 1 cm optical path (L) were used. Spectra were analyzed using the Spectra Analysis program contained in Spectra Manager v2 software. The protein concentration was calculated as follows: C = (absorbance_280nm_-absorbance_350nm_)/(ε×L). The molar extinction coefficient (ε) used was 1.43547 mL/mg.cm. The mass concentration was converted to amount of substance concentration (mol/L) using the molecular weight value corresponding to a mAb of 150 kg/mol.

### Circular dichroism spectroscopy

CD spectroscopy was used to analyze both the secondary structure (far-UV CD spectra) and the conformational features of the tertiary structure (near-UV CD spectra). It was measured on a Jasco J-1500 spectro-polarimeter (Jasco Corporation, Japan) previously calibrated with d-10-camphorsulfonic acid and equipped with Jasco PTC-510 Peltier temperature controller and mini- Jasco MCB-100 water circulation bath. The far-UV CD and near-UV CD spectra were obtained in the intervals of 190–250 nm and 250–350 nm, in quartz cuvettes with a path length of 0.1 and 1 cm, respectively. The spectra were collected by continuous scanning at 0.1 nm intervals, a scanning speed of 100 nm/min, a response time of 1 s, a bandwidth of 1 nm; 15 and 6 accumulations for far-UV CD and near-UV CD, respectively. The protein concentrations were 1 and 7 μmol/L for far-UV CD and near-UV CD, respectively. The baseline was corrected in all experiments using a protein-free PBS solution as control.

The ellipticity reading for each wavelength (θ_λ_) was presented as mean molar ellipticity by residue [θ]_λ_ according to the following equation: [θ]_λ_ = 100×θ_λ_/C×n×L. In this equation, θ_λ_ is the ellipticity (mdeg); n, the number of amino acid residues; C, protein concentration (mmol/L) and L, the optical step (cm). The value of [θ]_λ_ was expressed in deg.cm^2^.dmol^-1^ [16].

The secondary structure content of the proteins was estimated by the analysis of the deconvolution of the spectra obtained from the far-UV CD according to the CONTIN-LL algorithm [17, 18]. For this measure, the base of SP175 reference protein data on the Dichroweb Internet server (*http*:*//dichroweb*.*cryst*.*bbk*.*ac*.*uk/html/home*.*shtml*) [19, 20] was used. The influence of differences in protein concentration between batches was minimized in the representation of the spectra by a scaling factor by mean of the DichroMatch website (*http*:*//pcddb*.*cryst*.*bbk*.*ac*.*uk/home*.*php*) [21].

### Thermal denaturation temperature to CD

The Tm of thermal denaturation was determined by varying the absorbance value in the far-UV CD spectrum at 205 nm with temperature increases [22]. Far-UV CD absorption spectra were recorded in the range of 190–250 nm with quartz cuvettes of 0.1 cm optical path. A Jasco 1500 spectropolarimeter (Jasco Corporation, Japan) previously calibrated with d-10-camphorsulfonic acid was used and the protein concentration was 1 μmol/L. The spectra were collected by continuous scanning at 0.1 nm intervals, a scanning speed of 100 nm/min, a response time of 1 s, a bandwidth of 1 nm and 15 accumulations. The baseline was corrected with protein-free PBS solution.

Thermal studies were conducted by raising the temperature in 5°C intervals from 25 to 95°C, with this purpose it was used a Jasco PTC-510 Peltier temperature controller and Jasco MCB-100 water circulation mini-bath. Transition curves were normalized for the fraction of the folded protein using the Origin-v8.0 program (Origin Lab Corporation, USA) by the standard equation: % *CDdenaturation* = ((*θ*_25_−*θ_temp_*)/(*θ*_25_−*θ*_95_))×100. In this equation, θ_25_ and θ_95_ represent the ellipticity values for fully folded and unfolded species, respectively; θ_temp_ represents the ellipticity observed at 205 nm. The values of Tm were calculated as the temperature at which the maximum of the first derivative of the rate of ellipticity change at 205 nm against the temperature is observed. This estimation was made by a non-linear adjustment by the Boltzmann method, where the value of the inflection point of the curve corresponds to the value of Tm.

### Intrinsic tryptophan fluorescence

Fluorescence spectra were recorded on a Jasco-1500 spectro-polarimeter (Jasco Corporation, Japan) equipped with a Jasco FMO 522 fluorescence monochromator (Jasco FDT-538 fluorescence detector), Jasco PTC-510 Peltier temperature controller and mini-bath of water circulation Jasco MCB-100. The intrinsic fluorescence emission spectra of proteins were measured in the range of 300–450 nm, every 1 nm, with 1 sec response time and 1 accumulation; after excitation at 295 nm, to obtain fluorescence spectra derived from the tryptophan (Trp) residues [23]. A bandwidth of 5 and 10 nm was used for excitation and emission, respectively. A protein concentration of 0.3 μmol/L as well as quartz cuvettes of 10 mm optical path were used. The baseline was corrected in all experiments using protein-free PBS solution.

Changes in the intrinsic fluorescence emission of Trp residues located in different microenvironments were measured after the addition of increasing amounts of acrylamide as a water-soluble attenuator [23]. The acrylamide concentrations ranged from 50 to 400 mol/L [24], recording the emission spectra every 50 mmol/L of acrylamide with a Jasco ATS-530 automatic titrator. Three replicates of each mAb were evaluated, under agitation, at 25°C and 120 sec response time. The ratio of acrylamide absence (F_0_) and presence (F_AA_) fluorescence intensities were determined for each acrylamide concentration ([AA]) and evaluated after excitation at 295 nm. The relative exposure of Trp residues in mAbs structure were estimated from the Stern Volmer constant (Ksv). This constant is obtained from the linear regression of the experimental results according to the following equation F0FAA=1+Ksv×[AA] [13, 23, 25].

## Results

### Desalination of mAbs by molecular exclusion chromatography

**Fig 2:**
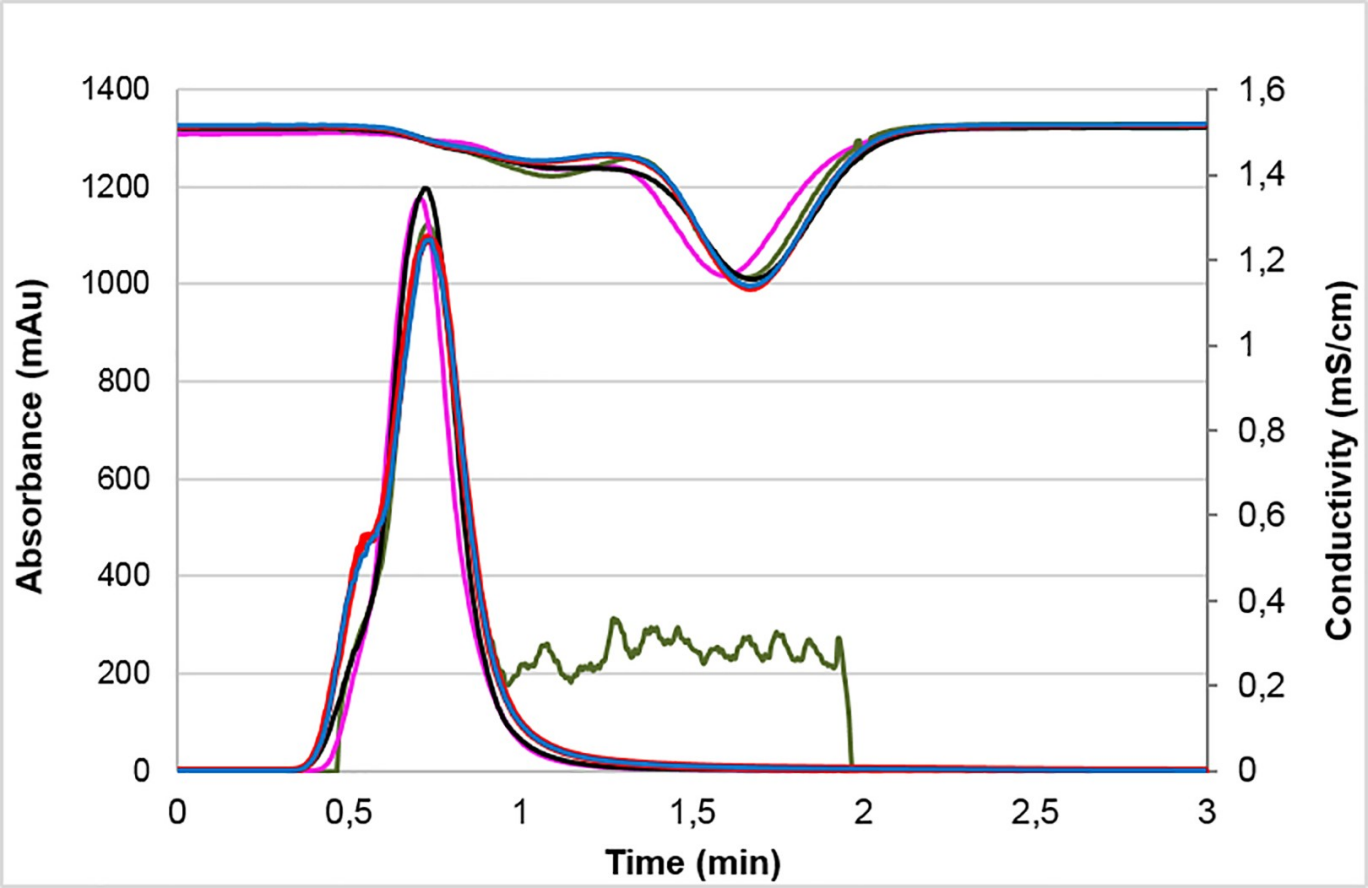
Molecular exclusion chromatographic profiles of 5G4 mAb and Herceptin. Absorbance at 280 nm (mAu) and conductivity (mS/cm) against elution time for different batches evaluated are shown. 5G4 mAb batch 1 (black), 5G4 mAb batch 2 (red), 5G4 mAb batch 3 (blue), Herceptin batch 1 (green) and Herceptin batch 2 (pink).

In order to eliminate the non-protein molecules present in the mAbs formulation, a buffer change to phosphate-buffered saline (PBS) was firstly performed using molecular exclusion chromatography (S2 Fig). Molecular exclusion chromatograms constructed for each batch of 5G4 mAb and Herceptin are shown in (Fig 2).

### Determination of 5G4 mAb secondary structure by far- ultraviolet (UV) circular dichroism (CD)

**Fig 3:**
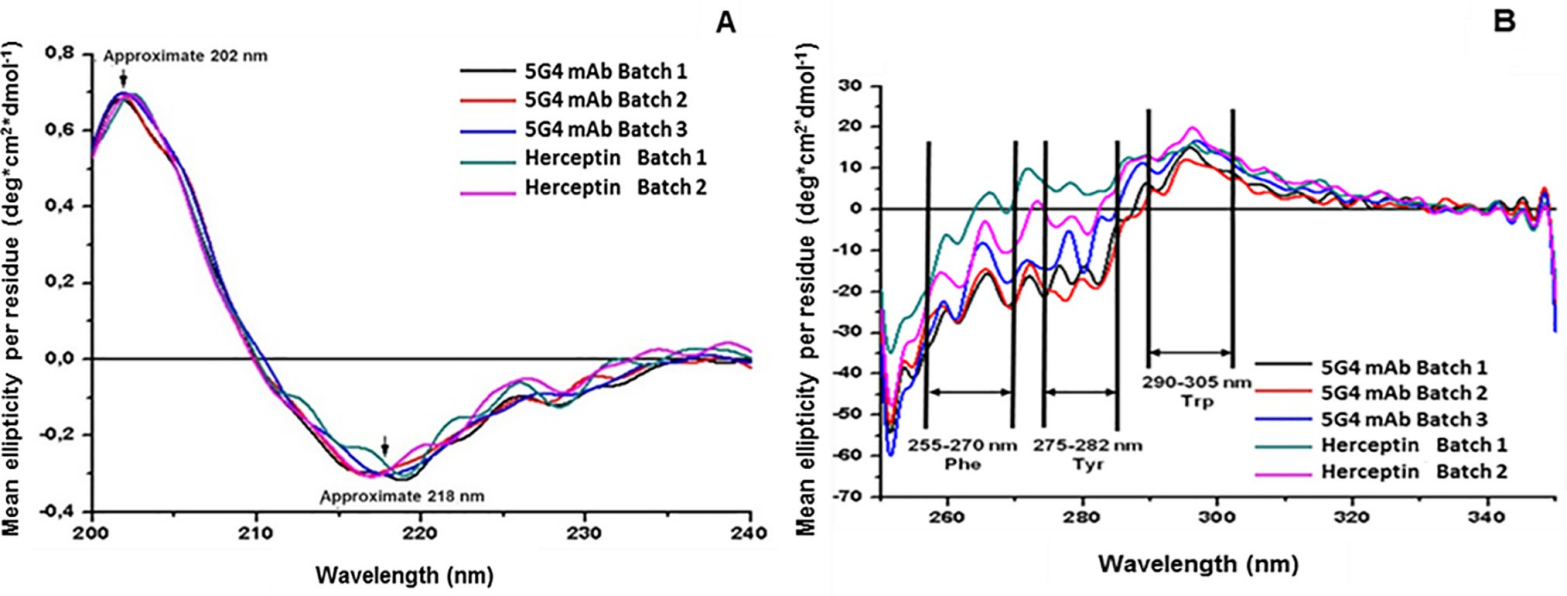
Far and near-UV CD spectra of 5G4 mAb and Herceptin in PBS solution, respectively. Different batches of each mAb are shown: 5G4 mAb batch 1 (black), 5G4 mAb batch 2 (red), 5G4 mAb batch 3 (blue), Herceptin batch 1 (green) and Herceptin batch 2 (pink). The mean ellipticity readings per residue (deg.cm^2^.dmol^-1^) for each wavelength at 25°C. **A**: Far-UV CD spectra of 5G4 mAb and Herceptin in PBS solution. The arrows indicate the positive band at about 202 nm and the negative band at about 218 nm. **B:** Near-UV CD spectra of 5G4 mAb and Herceptin in PBS solution. The arrows indicate the wavelength ranges corresponding to the signals of the side chains of Phe, Tyr and Trp.

The secondary structure of 5G4 mAb (in PBS) was determined from the far-UV CD analysis of the (200 to 240 nm) spectra. Fig 3A shows the CD spectra of 5G4 mAb in comparison with Herceptin. The spectra were very similar and showed the traits of proteins with high β-sheet content, which is characterized by a positive band around 202 nm and a negative band at approximately 218 nm (S1 Table). In order to establish whether the 5G4 mAb and Herceptin, produced in different cell lines, have the same conformational characteristics, the content of secondary structures was determined using the Dichroweb internet server and the CONTIN-LL algorithm. The deconvolution analysis of the CD spectra obtained in the far-UV shows similar percentages of α-helices, β-sheets and disordered structures for both antibodies (Table 1). A predominant occurrence of β-sheets structure was also confirmed.

**Table 1:** Percentage of secondary structures of 5G4 mAb and Herceptin by DichroWeb.

Secondary Structures (%)	5G4 mAb				Herceptin		
	Batch 1	Batch 2	Batch 3	Mean ± SD	Batch 1	Batch 2	Mean ± SD
α-helices	6.0	6.0	7.4	6.5 ± 0.8	6.2	6.0	6.1 ± 0.1
β-sheets	56.1	56.0	53.7	55.2 ± 1.4	55.5	55.9	55.7 ± 0.3
Disordered structures	37.9	37.4	39.0	38.1 ± 0.8	38.4	38.2	38.3 ± 0.1
Nrmsd	0.172	0.163	0.181		0.158	0.182	

Nrmsd, Normalized root-mean-square deviation; SD, Standard deviation.

### Tertiary structure analysis of 5G4 mAb by near-UV CD

The information on the tertiary structure of 5G4 mAb was analyzed in the near-UV (250 to 350 nm) as shown in Fig 3B. The results for all batches of 5G4 mAb indicated a three-dimensional folding of proteins, due to the bands of absorption characteristics of the aromatic amino acids present in this spectrum region. Negative absorption bands were observed between 255 to 270 nm, corresponding to phenylalanine (Phe), which showed a fine structure in this wavelength range. Between 275 to 282 nm the absorption bands corresponded to tyrosine (Tyr), displaying one shoulder at greater wavelengths overlapping with the Trp bands. In the spectra, also a maximum absorption corresponding to the Trp residues were observed, which showed a fine structure between 290 to 305 nm. Similar results were obtained with Herceptin batches.

### Thermodynamic stability of 5G4 mAb by far-UV CD

**Fig 4:**
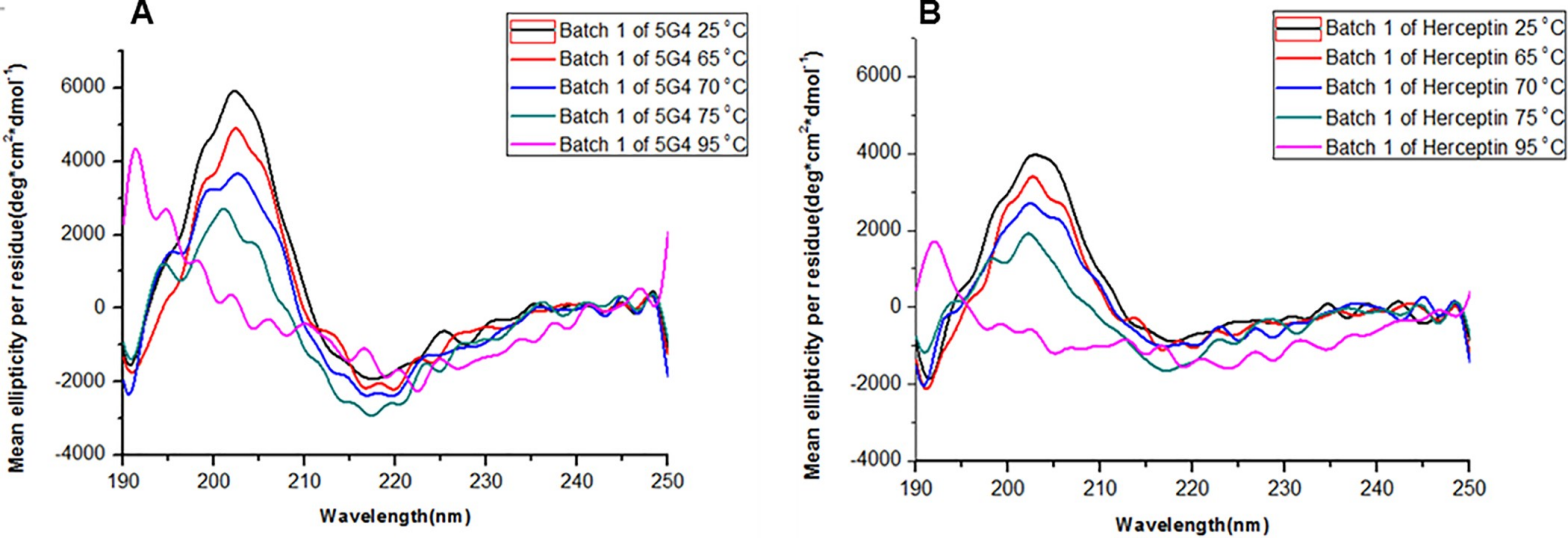
Thermal stability spectra by far-UV CD for 5G4 mAb and Herceptin in PBS solution. Batch 1 of 5G4 mAb (A) and batch 2 of Herceptin (B) are shown. Different temperatures of each mAb are shown: Temperature 25°C (black), temperature 65°C (red), temperature 70°C (blue), temperature 75°C (green) and temperature 95°C (pink).

The thermal stability of 5G4 mAb was determined from CD spectra in the far-UV region (190 to 250 nm) for a temperature range of 25 to 95°C, with intervals of 5°C. A loss of secondary structure from 70°C was observed for all bacthes of 5G4 mAb, due to a decrease of the mean ellipticity by residues at 202 and 218 nm, which are the typical bands of proteins predominantly structured in β-sheet. In addition, the wavelength corresponding to the zero crossing also varied, moving to lower values (Fig 4). The CD spectra obtained at this temperature range showed a comparable behavior for all 5G4 and Herceptin batches.

**Fig 5:**
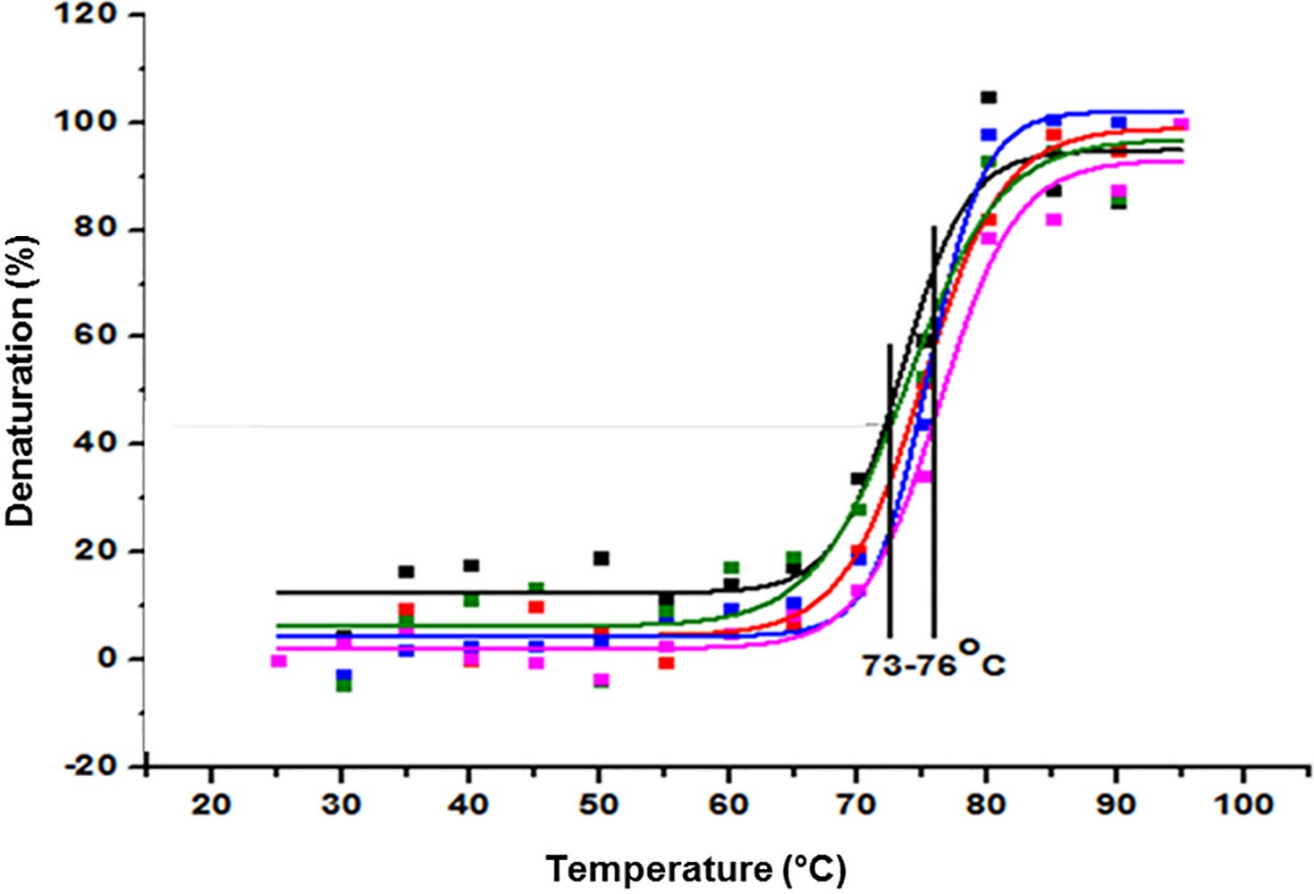
Thermal denaturation curves for 5G4 mAb and Herceptin in PBS solution. Different batches of the mAbs are shown. 5G4 mAb batch 1 (black), 5G4 mAb batch 2 (red), 5G4 mAb batch 3 (blue), Herceptin batch 1 (green) and Herceptin batch 2 (pink). The indicated region shows the temperature range corresponding to the Tm values obtained.

In order to calculate the melting temperature (Tm) values, the thermal denaturation at the wavelength of 205 nm was monitored according to Pérez et al., 2001. Fig 5 shows a similar thermal denaturation curves for 5G4 mAb and Herceptin batches. The values of Tm were in the range of 73 to 76°C with comparable values (~ 75°C) for both antibodies (Table 2).

**Table 2:** The temperature of melting for 5G4 mAb and Herceptin.

Sample	Tm (°C) ± SD	r^2^	Mean
**5G4 Batch 1**	73.33375 ± 1.03084	0.9491	74.59736
**5G4 Batch 2**	75.03291 ± 0.49424	0.9904	
**5G4 Batch 3**	75.42541 ± 0.45925	0.9864	
**Herceptin Batch 1**	73.79623 ± 1.16698	0.9575	75.0348
**Herceptin Batch 2**	76.27337 ± 0.60924	0.9845	

Tm, temperature of melting; SD, Standard deviation.

### Tertiary structure study of 5G4 mAb by intrinsic fluorescence measurement

**Fig 6:**
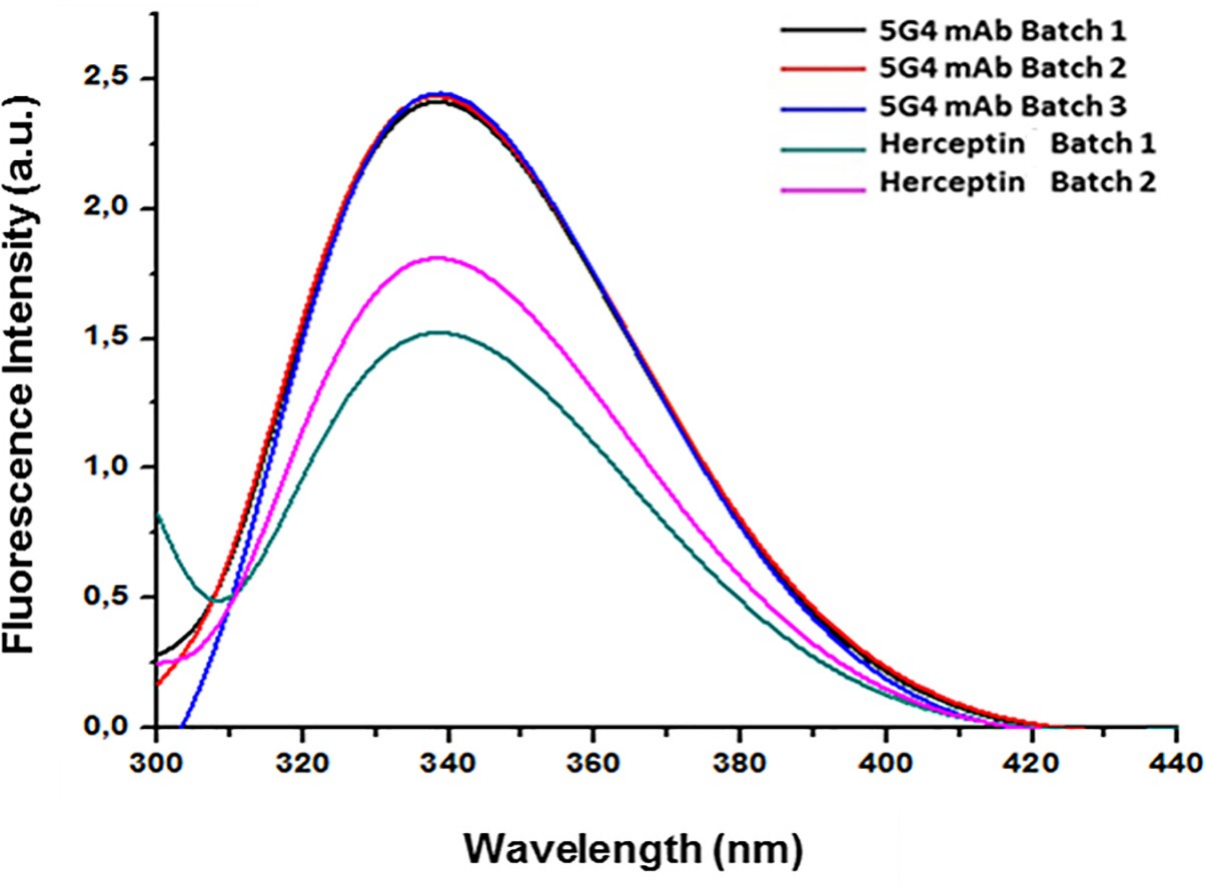
Fluorescence emission spectra for 5G4 mAb and Herceptin in PBS solution. Different batches of each mAb are shown. 5G4 mAb batch 1 (black), 5G4 mAb batch 2 (red), 5G4 mAb batch 3 (blue), Herceptin batch 1 (green) and Herceptin batch 2 (pink).

The conformational characteristics of 5G4 mAb were determined by fluorescence emission in the wavelength range (λ) of 300 to 450 nm, following selective excitation of Trp at 295 nm. In the Fig 6, the values of the emission maximums of the Trp and the intensity of the fluorescence for each lot of 5G4 mAb and Herceptin are shown. 5G4 mAb showed an increased emission intensity when compared with Herceptin (S3 Table). However, all the spectra showed similarity in wavelength of the emission maxima (λmax) of the Trp, with approximately 338 nm.

### 5G4 mAb conformational features by intrinsic fluorescence spectra of Trp

**Fig 7:**
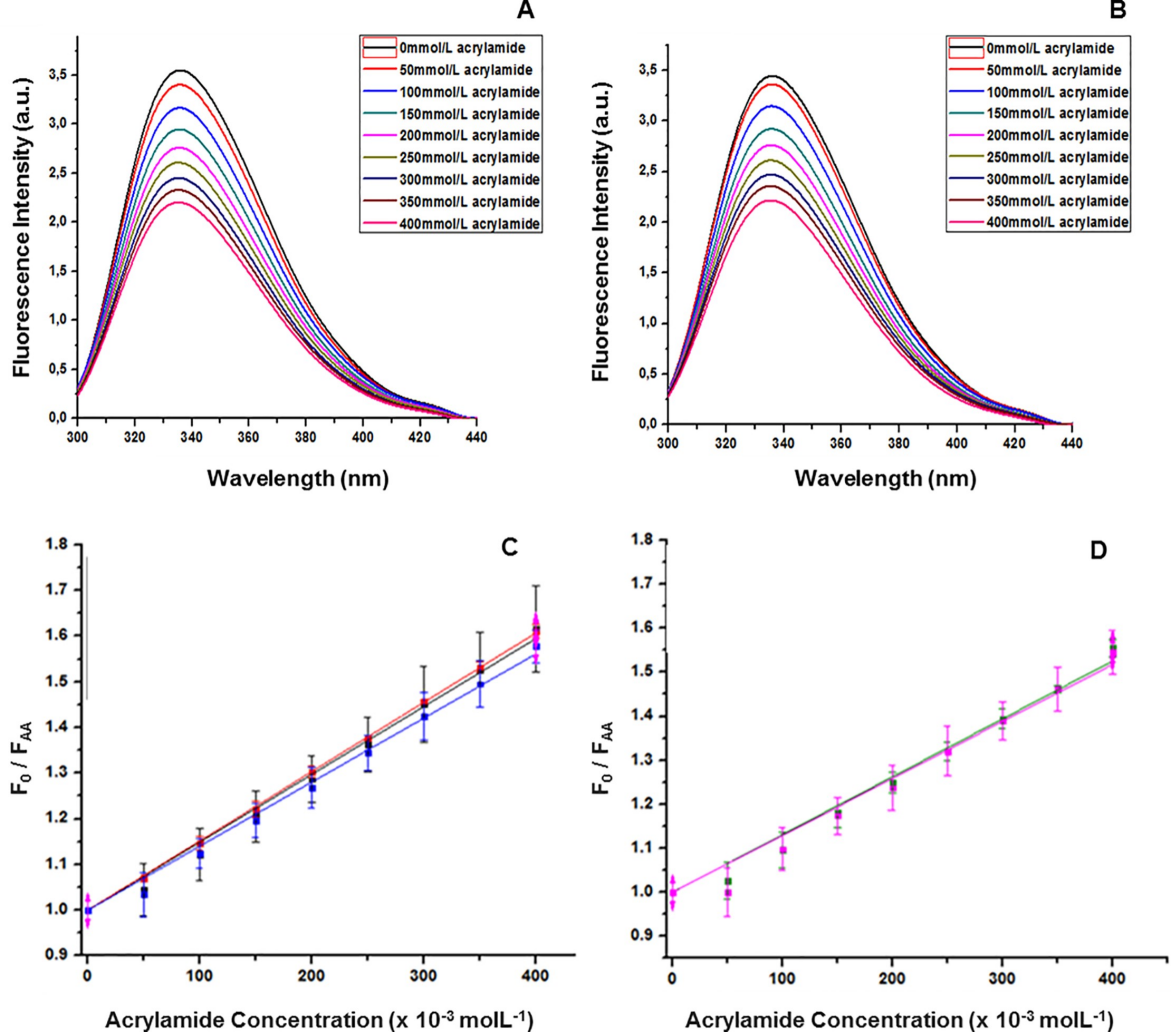
Degree of exposure of Trp residues. Fluorescence emission spectra of 5G4 mAb (A) and Herceptin (B) attenuated with different concentrations of acrylamide are shown. Black, red, light blue, yellow, dark blue, brown and pink colors spectra correspond to 0, 50, 100, 150, 250, 300, 350 and 400 mmol/L acrylamide. C and D: Stern Volmer representation for acrylamide attenuated 5G4 mAb and Herceptin. Different batches of each mAb are shown. 5G4 mAb batch 1 (black), 5G4 mAb batch 2 (red), 5G4 mAb batch 3 (blue), Herceptin batch 1 (green) and Herceptin batch 2 (pink).

Attenuation of intrinsic Trp fluorescence of 5G4 mAb was determined by measuring fluorescence intensity at increasing concentrations of acrylamide (50 to 400 mmol/L). The range of 300 to 450 nm was evaluated, after excitation at 295 nm, to obtain fluorescence spectra derived from Trp residues. Fig 7A and 7B show the fluorescence emission spectra, for one batch of 5G4 mAb and one for Herceptin. Then, the relative average exposure of Trp to the surface of both mAbs was calculated by mean of Ksv (Fig 7C and 7D). The values of Ksv for 5G4 mAb and Herceptin were 1.46 and 1.30 mol ^-1^ L, respectively (Table 3), which are indicative of a similar degree of exposure of Trp residues similar for both antibodies. However, a slight increase in the Ksv value for 5G4 mAb was evidenced when compared with Herceptin, in line with the slight increase observed in the fluorescence emission spectra.

**Table 3:** Stern-Volmer constant values (Ksv) for 5G4 mAb and Herceptin.

Sample	Ksv (mol ^-1^ L) ± SD	r^2^	Mean
5G4 Batch 1	1.45 ± 0.025	0.99	1.46
5G4 Batch 2	1.52 ± 0.005	0.99	
5G4 Batch 3	1.40 ± 0.035	0.99	
Herceptin Batch 1	1.31 ± 0.032	0.99	1.30
Herceptin Batch 2	1.29 ± 0.040	0.99	

Ksv, Stern Volmer constant; SD, Standard deviation.

## Discussion

In the present work, it is shown a conformational characterization study of a novel anti-HER2 mAb (5G4) by means of CD and Trp intrinsic fluorescence. The analysis of the secondary structure by far-UV CD showed typical spectral characteristics of β-sheets for 5G4 mAb, similar to those previously reported for Herceptin [26]. This result was corroborated when the percentage of secondary structures for both mAbs was compared using the DichroWeb tool (55 to 56% of β-sheets). Moreover, the three-dimensional folding of 5G4 mAb showed the appearance of absorption bands corresponding to the aromatic residues. The intensity of the Trp absorption signal for this mAb was comparable to Herceptin, although small differences for the Tyr and Phe residues were observed. These results are in correspondence with obtained by other authors for Herceptin [9].

The thermal stability evaluated by far-UV CD showed values of Tm between 73 to 76°C for both 5G4 mAb and Herceptin, indicating a similar folding and showing resistance to high temperatures, as previously described for Herceptin and other mAbs [27, 28]. In addition, the emission spectra of Trp by fluorescence for 5G4 mAb showed a higher emission intensity respect to Herceptin, indicative of an increasing in the quantum emission efficiency of this chromophore. Furthermore, a slight increase in the Ksv value of 5G4 mAb was observed when compared with Herceptin. This fact could be in correspondence with the slight augment in the fluorescence emission spectra detected for 5G4 mAb. However, all spectra of both mAbs showed similarity in the wavelength of the emission maximums of the Trp, suggesting no differences in their conformational environments.

A possible explanation for the slight increase in the values of Ksv and intensity of the spectra of 5G4 mAb with respect to Herceptin could be related to a self-attenuation effect on some Trp by the polar functional groups present in the amino acid side chains in their conformational environment. Besides, this behavior could be related with the differences in the cell lines used for the production of 5G4 mAb and Herceptin. These systems could introduce slightly different glycosylation patterns able to attenuate the effect of the polar chemical groups or modify the degree of exposure of some Trp residue in their proximity [23]. However, the amino acid sequence of light chain for 5G4 mAb showed a 100% of coincidence with Herceptin, while for the high chain only 98% of coincidence was found, characterized by the existence of two conservative amino acid changes (glutamic by aspartic and methionine by leucine). Nevertheless, only the first of these changes occurs between amino acids with attenuating capacity [23].

It is known, the binding properties (e.g. recognition and affinity) of anti-HER2 mAbs is an important factor in order to induce any mechanism of action. These properties are determined in part by the three-dimensional folding of these molecules. Despite the small differences found in the values of the Ksv and fluorescence emission intensity for 5G4 mAb respect to Herceptin, it is expected that these differences are unable to significatively reduce the biological activity of 5G4 mAb. In line with this, *Blanco et al*. recently reported similar binding properties of 5G4 mAb when compared with Herceptin. Moreover, preliminary studies showed both the capacity of 5G4 mAb to induce antibody-dependent cellular cytotoxicity (ADCC) and inhibition of cell proliferation in SK-BR-3 cells (HER2-positive cells), non-inferior to those obtained with Herceptin [29]. However, further experiments are necessary in order to confirm these results.

In summary, 5G4 mAb showed similar conformational characteristics when compared with Herceptin (reference product). The composition of secondary structure as well as the thermal stability of these mAbs were comparable. The similar thermal stability suggests a three-dimensional folding and high thermodynamic stability for 5G4 mAb. However, our data revealed potential differences concerning the degree of exposure of their aromatic residues. In this sense, experiments to X-ray crystallography and mass spectrometry are strongly recommended. Moreover, studies to demonstrate the specificity and recognition affinity for the subdomain IV of the extracellular domain of HER2 of 5G4 mAb are ongoing in our center.

## Supporting information

S1 Fig Sequence by MALDI and ESI of Herceptin and 5G4 mAb. (PDF)

S2 Fig Far UV-CD spectra of 5G4 mAb and Herceptin in the formulation solution. (PDF)

S1 Table Wavelengths of the positive and negative bands by far-UV CD spectra. (PDF)

S2 Table Position of the absorption bands by near-UV CD spectra of the 5G4 mAb and Herceptin. (PDF)

S3 Table Maximum of fluorescence emission of the tryptophan for 5G4 mAb and Herceptin. (PDF)

## Abbreviations

a.u: Arbitrary units
ADCC: Antibody-dependent cellular cytotoxicity
CD: Circular dichroism
CHO: Chinese Hamster Ovary cells
EMA: European Medicines Agency
FDA: Food and Drug Administration of the United States of America
FPLC: Fast Performance Liquid Chromatography
HER2: Epidermal growth factor receptor type 2
Ksv: Stern Volmer constant
mAb: Monoclonal antibody
mAu: arbitrary milliunits
Nrmsd: Normalized root-mean-square deviation
NSO: Non-secreting murine myeloma cells
PBS: Phosphate-buffered saline
Phe: Phenylalanine
SD: Standard deviation
Tm: Melting temperature
Trp: Tryptophan
Tyr: Tyrosine
UV: Ultraviolet
