# Supplementary material for: Conformational characterization of a novel anti-HER2 candidate antibody
Source: PLoS One. 2019 May 9;14(5):e0215442. doi: 10.1371/journal.pone.0215442 (PMC6508720; doi:10.1371/journal.pone.0215442)
Supplement: S1 Fig — (PDF) [file pone.0215442.s001.pdf]

A: Proteolytic degradation by trypsin, chymotrypsin, Lys-C and Asp-N enzymes of the heavy chain of Herceptin®. 97.1% coverage

EVQLVESGGGLVQPGGSLRLSCAAS**GFNIKDTY**IHWVRQAPGKGLEWVAR**IYPTNGYT**RYADSVKGRF
TISADTSKNTAYLQMNSLRAEDTAVYYC**SRWGGDGFYAMDY**WGQGTLVTVSSASTKGPSVFPLAPSSK
STSGGTAALGCLVKDYFPEPVTVSWNSGALTSGVHTFPAVLQSSGLYSLSSVVTVPSSSLGTQTYICN
VNHKPSNTKVDKKVEPKSCDKTHTCPPCPAPELLGGPSVFLFPPKPKDTLMISRTPEVTCVVVDVSHE
DPEVKFNWYVDGVEVHNAKTKPREEQYNSTYRVVSVLTVLHQDWLNGKEYKCKVSNKALPAPIEKTIS
KAKGQPREPQVYTLPPSR**E**E**M**TKNQVSLTCLVKGFYPSDIAVEWESNGQPENNYKTTPPVLDSDGSFF
LYSKLTVDKSRWQQGNVFSCSVMHEALHNHYTQKSLSLSPGK

B: Proteolytic degradation by trypsin, chymotrypsin, Lys-C and Asp-N enzymes of the heavy chain of 5G4 mAb. 96.0% coverage

EVQLVESGGGLVQPGGSLRLSCAA**SGFNIKDTY**IHWVRQAPGKGLEWVAR**IYPTNGYT**RYADSVKGRFT
ISADTSKNTAYLQMNSLRAEDTAVYYC**SRWGGDGFYAMDY**WGQGTLVTVSSASTKGPSVFPLAPSSKST
SGGTAALGCLVKDYFPEPVTVSWNSGALTSGVHTFPAVLQSSGLYSLSSVVTVPSSSLGTQTYICNVNH
KPSNTKVDKKVEPKSCDKTHTCPPCPAPELLGGPSVFLFPPKPKDTLMISRTPEVTCVVVDVSHEDPEV
KFNWYVDGVEVHNAKTKPREEQYNSTYRVVSVLTVLHQDWLNGKEYKCKVSNKALPAPIEKTISKAKGQ
PREPQVYTLPPSR**D**E**L**TKNQVSLTCLVKGFYPSDIAVEWESNGQPENNYKTTPPVLDSDGSFFLYSKLT
VDKSRWQQGNVFSCSVMHEALHNHYTQKSLSLSPGK

S1 Fig: Sequence by MALDI and ESI of Herceptin and 5G4 mAb. The proteolytic degradation of the heavy chain for both antibodies is observed. In red: mapped sequence, in black: sequence without mapping and highlighted in red: the CDRs. A and B: Proteolytic degradation by trypsin, chymotrypsin, Lys-C and Asp-N enzymes of the heavy chain of Herceptin and 5G4 mAb, respectively.
