# Supplementary material for: Conformational characterization of a novel anti-HER2 candidate antibody
Source: PLoS One. 2019 May 9;14(5):e0215442. doi: 10.1371/journal.pone.0215442 (PMC6508720; doi:10.1371/journal.pone.0215442)
Supplement: S2 Fig — (PDF) [file pone.0215442.s002.pdf]

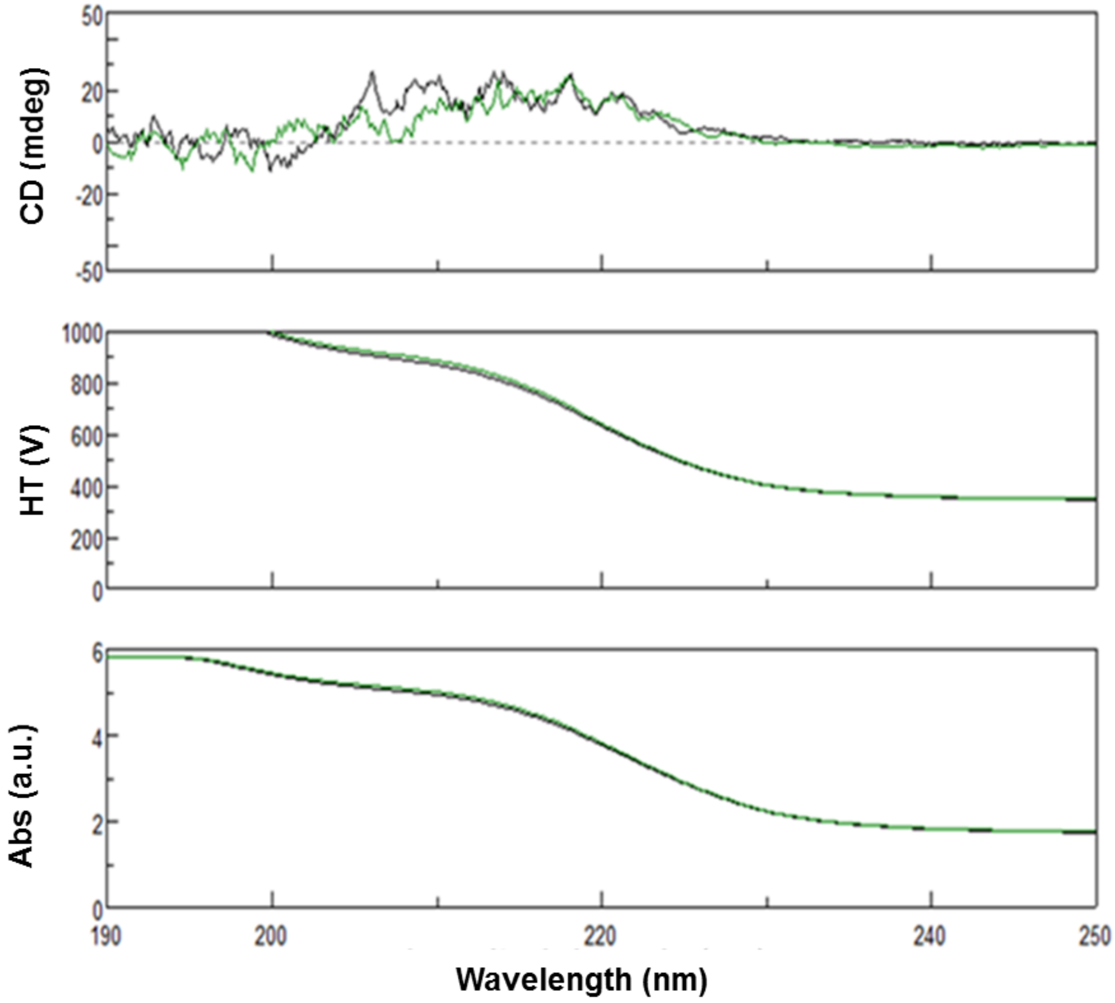

S2 Fig: Far UV-CD spectra of 5G4 mAb and Herceptin in the formulation solution. One batch of each mAb is shown: Herceptin (green) and 5G4 mAb (black). The readings of circular dichroism (CD), high tension (HT) and absorbance (Abs) for each wavelength are observed.
