# Supplementary material for: Conformational characterization of a novel anti-HER2 candidate antibody
Source: PLoS One. 2019 May 9;14(5):e0215442. doi: 10.1371/journal.pone.0215442 (PMC6508720; doi:10.1371/journal.pone.0215442)
Supplement: S1 Table — (PDF) [file pone.0215442.s003.pdf]

S1 Table: Wavelengths of the positive and negative bands by far-UV CD spectra.

Batches	β-Sheets wavelength characteristics (nm)	
5G4 mAb Batch 1	201.8	218.8
5G4 mAb Batch 2	201.8	216.7
5G4 mAb Batch 3	201.9	217.8
Mean ± SD	201.8 ± 0.1	217.8 ± 1.0*
Herceptin Batch 1	202.2	218.9
Herceptin Batch 2	202.5	217.0
Mean ± SD	202.4 ± 0.2	218.0 ± 1.3

Legend. SD, Standard deviation.
