# Supplementary material for: Conformational characterization of a novel anti-HER2 candidate antibody
Source: PLoS One. 2019 May 9;14(5):e0215442. doi: 10.1371/journal.pone.0215442 (PMC6508720; doi:10.1371/journal.pone.0215442)
Supplement: S2 Table — (PDF) [file pone.0215442.s004.pdf]

S2 Table: Position of the absorption bands by near-UV CD spectra of the 5G4 mAb and Herceptin.

Batches	Absorption maximum of the Trp (nm)
5G4 Batch 1	295.9
5G4 Batch 2	295.1
5G4 Batch 3	296.9
Mean ± SD	296.0 ± 0.9
Herceptin Batch 1	296.2
Herceptin Batch 2	296.2
Mean ± SD	296.2 ± 0

Legend. SD, Standard deviation; Trp, Tryptophan.
