# Supplementary material for: Conformational characterization of a novel anti-HER2 candidate antibody
Source: PLoS One. 2019 May 9;14(5):e0215442. doi: 10.1371/journal.pone.0215442 (PMC6508720; doi:10.1371/journal.pone.0215442)
Supplement: S3 Table — (PDF) [file pone.0215442.s005.pdf]

S3 Table: Maximum of fluorescence emission of the tryptophan for 5G4 mAb and Herceptin.

Batches	Emission maximum of the Trp (nm)	Intensity (a.u.)
5G4 mAb Batch 1	338.0	2.41
5G4 mAb Batch 2	338.0	2.43
5G4 mAb Batch 3	339.0	2.44
Mean ± SD	338.3 ± 0.6	2.43 ± 0.02
Herceptin Batch 1	339.0	1.52
Herceptin Batch 2	338.0	1.81
Mean ± SD	338.5 ± 0.7	1.67 ± 0.2

Legend. SD, Standard deviation; Trp, Tryptophan.
